# The Use of Intact Fish Skin Grafts in the Treatment of Necrotizing Fasciitis of the Leg: Early Clinical Experience and Literature Review on Indications for Intact Fish Skin Grafts

**DOI:** 10.3390/jcm12186001

**Published:** 2023-09-16

**Authors:** Philip Dueppers, Roland Bozalka, Reinhard Kopp, Anna-Leonie Menges, Benedikt Reutersberg, Claudia Schrimpf, Francisco Jose Moreno Rivero, Alexander Zimmermann

**Affiliations:** 1 Department of Vascular Surgery, University Hospital Zurich (USZ), University of Zurich (UZH), Raemistrasse 100, CH-8091 Zurich, Switzerland; roland.bozalka@usz.ch (R.B.); reinhard.kopp@usz.ch (R.K.); anna-leonie.menges@usz.ch (A.-L.M.); benedikt.reutersberg@usz.ch (B.R.); claudia.schrimpf@usz.ch (C.S.); alexander.zimmermann@usz.ch (A.Z.); 2 Tissue Viability Service (Wound Care), University Hospital Zurich (USZ), Raemistrasse 100, CH-8091 Zurich, Switzerland; franciscojose.morenorivero@usz.ch

**Keywords:** intact fish skin, fish skin grafts, necrotizing fasciitis, wounds, chronic wounds, gangrene, skin substitutes, wound healing, negative pressure wound therapy, skin transplantation

## Abstract

Background: Necrotizing fasciitis (NF) is a serious infectious disease that can initially place the patient’s life in danger and, after successful surgical and antibiotic treatment, leaves extensive wounds with sometimes even exposed bones and tendons. Autologous skin grafts are not always possible or require adequate wound bed preparation. Novel intact fish skin grafts (iFSGs; Kerecis^®^ Omega3 Wound, Kerecis hf, Isafjördur, Iceland) have already shown their potential to promote granulation in many other wound situations. Faster wound healing rates and better functional and cosmetic outcomes were observed due to their additionally postulated anti-inflammatory and analgesic properties. Therefore, iFSGs may also be essential in treating NF. We present our initial experience with iFSGs in treating leg wounds after NF and review the literature for the current spectrum of clinical use of iFSGs. Case Presentations: We present two male patients (aged 60 and 69 years) with chronic or acute postsurgical extensive leg ulcers six weeks and six days after necrotizing fasciitis, respectively. Both suffered from diabetes mellitus without vascular pathologies of the lower limbs. A single application of one pre-meshed (Kerecis^®^ Graftguide) and one self-meshed 300 cm^2^ iFSG (Kerecis^®^ Surgiclose) was performed in our operation room after extensive surgical debridement and single circles of negative wound pressure therapy. Application and handling were easy. An excellent wound granulation was observed, even in uncovered tibia bone and tendons, accompanied by pain relief in both patients. Neither complications nor allergic reactions occurred. The patients received autologous skin grafting with excellent functional and cosmetic outcomes. Conclusions: iFSGs have the potential to play a significant role in the future treatment of NF due to the fast promotion of wound granulation and pain relief. Our experience may encourage surgeons to use iFSGs in NF patients, although high-quality, large-sized studies are still required to confirm these results. The observed effects of iFSGs on wounds associated with NF may be transferred to other wound etiologies as well.

## 1. Introduction

Necrotizing fasciitis (NF, syn. streptococcal gangrene, gas gangrene, suppurative fasciitis, Meleney’s gangrene, necrotizing erysipelas, and Fournier’s gangrene) is a rare, rapidly progressing, severe, and life-threatening infection of the subcutaneous fat tissue, muscle and fascia of usually the lower extremities, genitalia or perineum [1]. Its pathomechanism usually starts with a minor trauma or wound as the access route for bacteria, leading to a soft tissue infection with subsequent necrosis and potentially life-threatening conditions. NF can be classified as type 1, caused by mixed aerobic and anaerobic organisms (polymicrobial), often from enteral origin, or as type 2 in a mono-infection with most commonly streptococcus pyogenes or other species [2]. Timely diagnosis with early surgical debridement and antibiotic therapy according to bacterial culture is vital to prevent septicemia, infectious shock, multiple organ dysfunction syndrome or even death, which has been reported in up to 12–20% of cases [1]. Extensive open wounds usually remain with a substantial impairment of patients’ health-related quality of life [2]. The loss of subcutaneous tissue and fascia with often uncovered bones or tendons challenges the plastic reconstruction of these significant defects.

As a novel skin substitute, fish skin grafts have already shown their potential to promote wound granulation and epithelization in many in vitro, animal and clinical studies [3,4,5]. To date, only one certified fish skin graft, originating from the wild North Atlantic cod (*Gadus morhua*), is commercially available, while alternatives from other fish species are still being evaluated in premarket studies [3,4,5]. This acellular but structurally intact fish skin graft (iFSG; Kerecis hf, Isafjördur, Iceland) underwent a proprietary process to preserve the original extracellular matrix and chemical composition of the fish skin, including proteoglycans, glycoproteins, soluble collagen, elastin, laminin, fibronectin and lipids [6,7]. Furthermore, iFSGs are structurally and chemically comparable to human skin but carry many omega-3 polyunsaturated fatty acids, known for their anti-inflammatory potential [8,9,10,11]. The combination of the intact microporous structure of the fish skin’s extracellular matrix with a chemical similarity to human skin and additional omega-3 fatty acids are probably the most essential factors for clinically proven wound healing promotion. In contrast to synthetic materials, the absence of autoimmune reactions and cultural or religious barriers are further advantages [12,13,14,15,16]. Contraindications only involve a known allergy or other sensitivity to fish material. The iFSGs are available in several sizes, shapes and variations, including pre-meshed or granulated products in the U.S. market, while the European CE certification of those pre-meshed and granulated products, as well as of larger fish skin grafts, is still pending. The clinical spectrum of iFSGs has been reported in several case reports up to prospective randomized-controlled trials and involves typical and atypical chronic wounds and traumatic wounds, including burns in adult and pediatric patients [15,17,18,19,20,21,22,23,24,25,26,27,28,29,30,31,32,33,34,35].

The use of iFSGs to promote the healing of wounds due to NF has yet to be reported in the current literature. Therefore, this article aims to present our experience with iFSGs in the treatment of either a chronic as well as an acute postoperative wound after extensive surgical debridement due to NF. Further, we present the clinical use of the iFSGs reported in the current literature.

## 2. Methods

**Figure 1:**
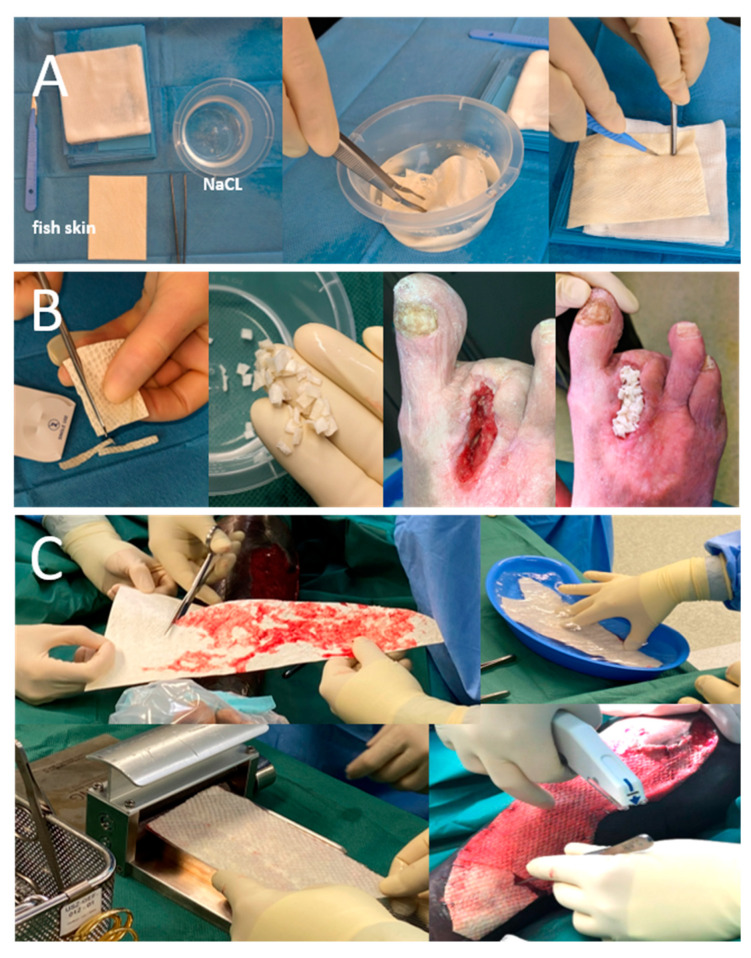
Different physician modifications of solid iFSGs: (**A**) Most usual graft fenestration using a surgical scalpel blade no. 11. (**B**) Graft shredding to achieve wound bed contact, even in deep wound beds, such as a non-healing toe amputation zone. (**C**) Intraoperative meshing of a cut-to-shape and hydrated large iFSG using a Zimmer air dermatome (Zimmer Biomet^®^, Warsaw, IN, USA) and fixation to the wound bed with a stapler.

Both presented patients officially agreed to the anonymous publication of their data and images according to the local requirements for case reports. Intact fish skin grafts, manufactured by Kerecis hf, Isafjördur, Iceland, were applied. First, applications were performed only in freshly debrided, clean, non-infected, or non-critically colonized wound beds (according to regularly performed morphological exams and clinical appearance). In the presented patients, we used meshed grafts when treated in the operating room (OR) and fenestrated grafts when treated in our dedicated outpatient wound center. The modification of solid iFSGs to achieve meshed (in the absence of pre-meshed products in one patient) or fenestrated grafts was performed (Figure 1A,C). All grafts were hydrated in a saline solution of 0.9% for two minutes, cut into shape to fit the wound area, and fixed with staplers or contact layers. The wounds were covered with an antiseptic paraffin gauze contact layer (Bactigras^®^, Smith & Nephew, London, UK) in our OR or silicone contact layers (Mepitel One^®^, Molnlycke, Goeteborg, Sweden) for subsequent dressings or re-applications in our outpatient wound center. Sterile compresses with a saline solution of 0.9% were applied to maintain a moist wound atmosphere. Secondary dressing changes were obtained every second day with the re-application of moist, sterile compresses according to the instructions for use. In the presented patients, we changed the complete dressing after five days while still in the hospital to react appropriately in case of complications, such as an infection or failed local exudation management. After discharge, complete dressing changes with superficial debridement and an evaluation of re-applications of iFSGs were performed after 7–14 days, depending on the wound situation.

For the narrative review on the clinical indications for iFSGs, a literature search using PubMed^®^ (pubmed.ncbi.nlm.nih.gov, accessed on 1 July 2023) with the terms “fish skin” was conducted. Studies on non-commercially available fish skin grafts were not considered for this review.

## 3. Case Presentations

### 3.1. Case 1: Chronic Leg Ulcer due to Necrotizing Fasciitis

**Figure 2:**
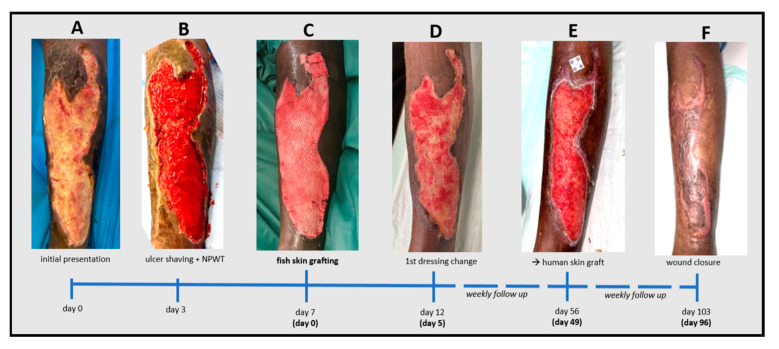
Timeline of the treatment of a chronic leg ulcer caused by necrotizing fasciitis supported by an intact fish skin graft (iFSG). After initial presentation (**A**) followed by ulcer shaving (**B**) and few days of negative pressure wound therapy a physician-meshed 300cm^2^ iFSG (Kerecis^®^ Graftguide) was applied (**C**). Wound granulation was excellent after 5 days (**D**) but due to a slow epithelialization over time (**E**), the wound was finally closed using a split skin graft with complete closure 3 months after iFSG transplantation (**F**).

A 69-year-old man, who had just returned from Sri Lanka after suffering from NF more than six weeks ago, presented to our emergency unit with a sizeable chronic ulcer involving almost the entire front of his left lower leg (Figure 2A), necessitating immediate treatment. In Sri Lanka, the wound was surgically debrided and treated with multiple antibiotics. Detailed medical reports were not available.

Upon presentation, the patient reported only mild pain at the ulcer site (numeric pain rating scale (NRS) at rest/during movement = 1/2). Relevant comorbidities were limited to poorly controlled type 1 diabetes (HbA1c 7.1%). Vascular pathologies were ruled out by ultrasound. The systemic inflammation parameters were slightly elevated (leucocytes 12.30 g/L; CRP 9.5 mg/L). Antibiotic therapy with a combination of amoxicillin and clavulanic acid was initially continued. The wound was temporarily covered with a silicon layer and sterile compresses, and the patient was admitted as an inpatient. Emergency surgical wound care was not required at this time. After three days, a deep surgical debridement with ulcer shaving using the Zimmer Biomet^®^ air dermatome (Zimmer Biomet, Warsaw, IN, USA) was performed, and an additional single circle of negative pressure wound therapy (NPWT) was applied. The wound was about 0.5 cm deep without uncovered bones or tendons but with well-perfused tissue (Figure 2B). Another four days later, the wound bed was prepared by superficial debridement in the OR and then covered with a physician-meshed 300 cm^2^ iFSG (Kerecis^®^ Surgiclose) (Figure 1C and Figure 2C). Antibiotic therapy was discontinued due to the absence of bacteria in the microbiological exams. The first complete dressing change was scheduled after five days, demonstrating impressive wound granulation and a slightly reduced and more superficial wound size (Figure 2D). We refrained from any further debridement and applied absorbent foam dressings. The patient was dismissed six days after the iFSG application. Weekly follow-up visits in our outpatient wound center showed increasing epithelialization from the wound edges, although this was not as impressive as the granulation, which continuously progressed over time. Re-application of the iFSG was not performed during the follow-up. After seven weeks, the wound had decreased in size, the upper edge was closed, and the wound was very well granulated (Figure 2E). However, the wound showed significant exudation and was colonized by pseudomonas aeruginosa without signs of local or systemic infection. The wound was mechanically debrided in our outpatient wound center and dressed with silver-impregnated hydrofiber and absorbent cellulose pads. However, it was time to decide whether to continue with conservative therapy, regular dressing changes, and maybe a re-application of an iFSG or perform autologous skin grafting. Therapeutic options were discussed with the patient, who preferred autologous skin grafting as the fastest way to close the wound. Complete wound closure was achieved 3.5 months (103 days) after the initial presentation and three months (96 days) after the iFSG application (Figure 2F). The patient has completely returned to his daily routine without limitations.

### 3.2. Case 2: Postsurgical Wound after Acute Necrotizing Fasciitis

**Figure 3:**
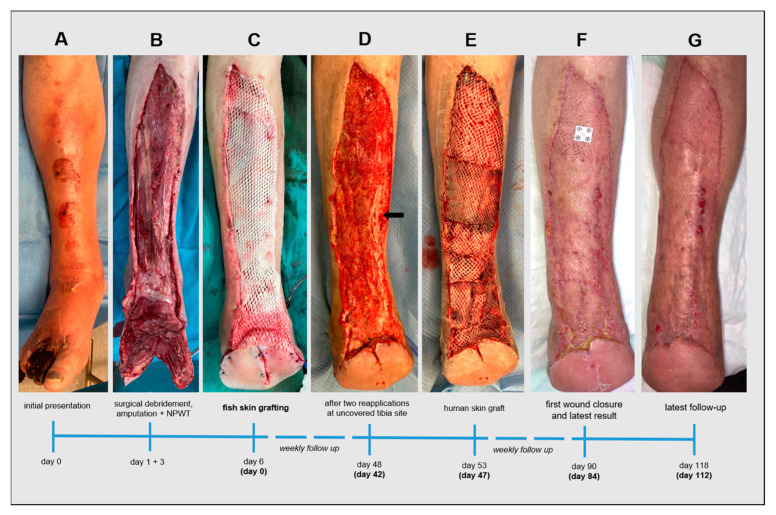
Timeline of the treatment of a postsurgical acute wound supported by an intact fish skin graft (iFSG) after extensive tissue loss due to necrotizing fasciitis. After initial presentation (**A**) and immediate surgical extensive debridement, forefoot amputation (**B**) and few days of negative pressure wound therapy a pre-meshed 300cm2 iFSG (Kerecis^®^ Graftguide) was applied onto the remaining wound bed (**C**). After six weeks and two isolated reapplications on an uncovered tibia site, the wound bed granulation was excellent (**D**) and a split skin graft transplantation was performed (**E**) to achieve complete wound closure after another 5 weeks (**F**) and an aesthetically and functionally satisfying late outcome (**G**).

**Figure 4:**
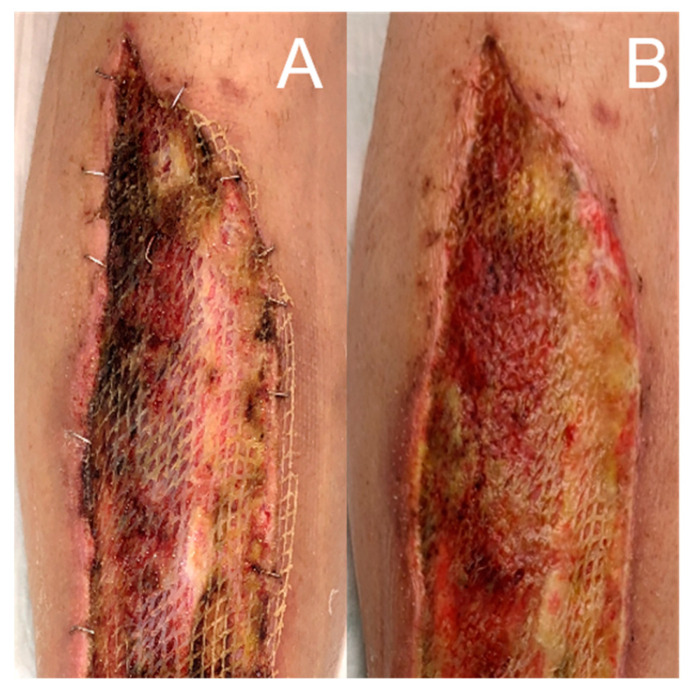
Salvage of a dry iFSG in a low exudating wound five days after its application (**A**) using hydrogel (Prontosan^®^, B. Braun Medical, Bethlehem, PA, USA) with a good result, graft dilution and incorporation and wound bed granulation after another five days (**B**).

**Figure 5:**
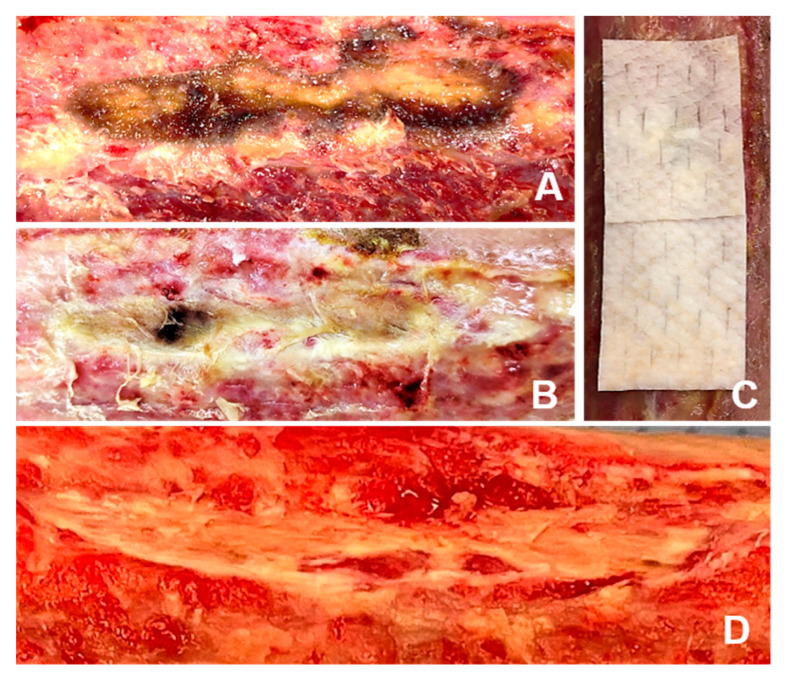
Treatment of a nearly uncovered tibia area with two applications of iFSGs at two-week intervals supported by hydrogel (Prontosan^®^, B. Braun Medical, Bethlehem, PA, USA) and dressed with a silicone interface and sterile compresses (**A**–**C**). No local debridement was performed, and the periosteum was left untouched. Due to the local progressive granulation (**D**), the patient was scheduled for final meshed autologous skin grafting.

A 60-year-old man presented to our emergency unit with necrotic toes, a severe anterior right lower leg infection with edema, and increased pain (NRS = 4/4) that required urgent treatment (Figure 3A). He reported that these findings had started and rapidly progressed after a foot trauma three days before. An oral antibiotic therapy without success followed the emergent presentation at his family doctor. Blood exams revealed a formerly unknown type 2 diabetes (HbA1c 13.1%) and strongly elevated inflammatory parameters (CRP 180 mg/dL; leucocytes 16.4 g/L). The patient’s body temperature was 38.1 °C. Empiric intravenous antibiotic therapy with piperacillin-tazobactam was initiated. Computer tomographic angiography demonstrated subcutaneous gas in the foot and the lower limb. There was no evidence of peripheral arterial occlusive disease. Due to his pre-septic condition and the suspicion of NF, the patient was immediately transferred to the OR. An emergent transmetatarsal Lisfranc amputation was performed, including excision of the infected cutaneous and subcutaneous areas and the underlying fascia. This was followed by NPWT. He was transferred to our peripheral ward postoperatively due to his stable hemodynamic condition. Two further operative revisions with surgical debridement, secondary Chopart amputation and changes in the NPWT dressing took place after one and three days (Figure 3B). Meanwhile, microbiological exams revealed a mix of staphylococcus aureus, enterobacter cloacae and enterococcus faecalis. In accordance with the defined antibiotic resistance, vancomycin was added to the antibiotic therapy. Histology from the intraoperatively excised samples from the cutis/subcutis and the fascia and the microbiological, clinical and radiological findings confirmed the preoperatively suspected diagnosis of NF type 1. The inflammatory parameters and body temperature normalized in due course. After six days, including a recurrent superficial debridement and disinfection of the wound bed, a 300 cm^2^ pre-meshed iFSG (Kerecis^®^ Graftguide) was applied (Figure 3C). The first complete dressing change after five days showed a very dry iFSG and wound bed. The graft was not diluted, slightly shrunk, and not incorporated with the wound bed (Figure 4A). To achieve and maintain a moist wound atmosphere and to support graft incorporation, hydrogel (Prontosan^®^, B. Braun Medical, Bethlehem, PA, USA) was applied directly onto the iFSG (without NPWT continuation), leading to an impressively improved result with graft graft salvage and finally beginning wound granulation granulation after another five days (Figure 4B). The patient was transferred to a rehabilitation center 18 days after admission (12 days after iFSG) and was followed up weekly at our outpatient wound center. Most of the wound’s bed demonstrated a fast granulation without infection or allergic reaction. An exposed tibia area, which was only covered by the periosteum, was treated with two re-applications of a 3 × 7 cm iFSG (Kerecis^®^ Omega3 Wound) and hydrogel (Prontosan^®^, B. Braun Medical, Bethlehem, PA, USA) and dressed with a silicone contact layer and sterile compresses in a two-week interval (Figure 5). The periosteum was left untouched, and the area showed good granulation. There was no evidence of osteomyelitis. The rest of the wound was dressed with a thin foam dressing. Along with our experience from case 1, we performed autologous skin grafting. We achieved a nearly complete wound closure five weeks later (Figure 3F) and an excellent result four months after the initial presentation (Figure 3G). The former wound area was soft with resistant superficies. Focal hypergranulation at the tibia bone site was treated with a silver nitrate stick. Today, the patient can walk with crutches, and rehabilitation is ongoing without functional deficits of the lower limb besides Chopart amputation.

## 4. Discussion and Literature Review

Two patients with chronic or acute extensive leg ulcers due to NF and treated with iFSGs are presented. Several experiences with the use of iFSGs in wounds caused by multiple etiologies have been published (Table 1); however, this is, to the best of our knowledge, the first report on the successful treatment of both chronic and acute postoperative NF-related ulcers using iFSGs in the current literature.

Extensive tissue loss challenges the healing of wounds associated with NF. Large (musculocutaneous) flaps to reconstruct the entire defect are not always possible or available. The presence of uncovered bones or tendons often impedes immediate autologous skin grafting. Therefore, prior promotion of wound bed granulation and the coverage of critical structures is crucial to achieve complete wound closure and to prevent amputation in the case of leg ulcers. A common approach for wound bed granulation in these situations is NPWT, which we also applied for a short term in the presented patients. Another therapeutic option, especially in the acute phase of NF, is hyperbaric oxygen therapy; however, apart from its weak evidence, its lack of widespread availability limits its standard use in NF treatment [39]. In general, evidence of surgical treatment for NF is minimal [40]. A recent systematic review of seven studies comparing the standard of care (SOC) with NPWT for NF therapy showed a significant mortality reduction after NPWT [1]. However, the number of debridements (one to five), total length of hospital stay (14–96 days), and complication rate were comparable. The presented patients were discharged faster (6–18 days after iFSG) and continued outpatient treatment, contributing to the patient’s quality of life and cost-efficiency. In addition, functional and esthetic outcomes and the quality of the healed tissue might be improved with iFSGs compared with the SOC or NPWT, as the study results from deep dermal burns suggest [32]. Personal observations in those reported and patients treated by us for other pathologies with iFSGs, as well as the published experiences from other clinical applications, similarly indicate a reduction in pain and local infection [29,30,32]. However, the promising results of our experience should encourage other surgeons to use iFSGs for these extensive wounds. To evaluate if this holds true, larger-sized randomized-controlled trials (RCT) comparing the standard of care alone or NPWT with iFSGs in patients with NF should be conducted in the future.

Uncovered tendons or bones are always challenging in wound healing. The uncovered bone on patient 2 was successfully treated with multiple iFSG transplantations before split-skin grafting. Alternative materials for this purpose, such as a biodegradable temporizing matrix (Novosorb^®^ BTW, Polymedics, Denkendorf, Germany), have been described in the literature with equally good results [41,42]. Comparing studies should be conducted for a comparison of clinical effectiveness as well as for cost-effectiveness.

In addition to wound bed preparation, therapy of the underlying wound etiology is crucial for wound healing. In typical wound etiologies, such as chronic venous insufficiency (CVI) or peripheral arterial occlusive disease (PAOD) [43], this may include surgical vein stripping or closure as well as a re-establishment of venous blood flow in CVI secondary to post-thrombotic syndrome as well as surgical or conservative measures to maintain sufficient peripheral arterial perfusion and to prevent acute thromboembolic or chronic arterial occlusion in patients with PAOD or peripheral aneurysms [36,37,44,45,46]. In NF therapy, appropriate antibiotic management and the excision of infected and necrotic tissue, as was performed in both our patients, are the most important steps before wound healing can be initiated. This is especially important before applying iFSGs.

Techniques and modes of application of iFSGs vary depending on the treated wound morphology, treatment setting and preferences of the treating physician and wound care team. In patients with non-NF-associated wounds, we have also used wound closure strips or sutures for graft fixation or shredded the solid iFSGs to fill out deeper wound areas (Figure 1B). For outpatient management, a complete dressing change interval of 7–14 days has become an established routine in our department. Superficial debridement with facultative re-application of an iFSG is usually not performed earlier than 14 days.

Apart from NF therapy, iFSGs have already been used in a broad spectrum of clinical situations. Typical and commonly reported indications for iFSGs involve chronic wounds caused by peripheral arterial occlusive disease, chronic venous insufficiency or diabetes mellitus [15,17,18,19,20,21,22,23,24,38]. These reports also describe its successful use in uncovered tendons or bones, as in our second patient. Most of these experiences were published as case reports or series. However, one randomized-controlled trial comparing iFSGs versus the standard of care (SOC) in diabetic foot ulcers was recently published, while others are ongoing [20,21,22,47]. The multi-center RCT by Lantis and colleagues involving 102 patients has shown superior wound healing rates and wound area reduction over the SOC for diabetic foot ulcers treated with iFSGs (*p* = 0.0163) at 12 weeks [47]. The results of the European Horizon-funded multi-national, multi-center RCT (Odin trial; H2020-EU.3.; H2020-EU.2.1) and its French subgroup, the KereFish study, are anticipated to be published within this year [22]. Aside from the typical etiologies of chronic wounds, atypical wounds were successfully treated with iFSGs [15,33,34,35]. Etiologies included vasculitis, ischemic necrosis, necrotic angiodermatitis, calciphylaxis and hemophilia.

Other indications for iFSGs are acute traumatic wounds and burns [24,29,30,31,32,48]. RCTs on acute wounds showed faster healing rates for iFSGs compared to human amnion/chorion membrane (Epifix^®^, MiMedx Group, Marietta, GA, USA) or porcine small-intestine submucosa (Oasis^®^, Smith and Nephew plc, London, UK) [6,16]. In those studies, the participants involved were healthy volunteers; therefore, these study settings were only partially adaptable to clinical scenarios with elderly, multimorbid patients. In deep dermal partial-thickness burns, healing rates after a single application of iFSGs were significantly better than conventional care in a small retrospective cohort study [24]. As a secondary finding of this study, a prolonged application (10 days vs. 5 days after onset) showed significantly better wound healing rates. We generally share the same experience with our patients and leave the grafts on the wound for up to 14 days if no wound complication occurs. Wallner and colleagues reported on 12 patients with mixed dermal burn wounds, of which all initially received an enzymatic debridement with NexoBrid^®^ (MediWound Germany GmbH, Rüsselsheim, Germany). They compared the results of iFSGs with Suprathel^®^ (PolyMedics Innovations GmbH, Denkendorf, Germany) and autologous split-thickness skin grafting [32]. Wounds treated with iFSGs demonstrated accelerated wound healing, better pain relief, and improved functional and cosmetic outcomes. In another publication, partial thickness burns in two patients showed full epithelization two weeks after the iFSGs and did not require autologous skin grafting [29]. Reda and colleagues have even reported the treatment of full-thickness burns and blast areas during an armed conflict [48]. In the context of burn injuries, iFSGs have also been used as a dermal substitute for split-thickness graft donor sites with encouraging results and accelerated epithelialization up to a significantly faster healing time than the SOC [29,30,31]. Yoon et al. could additionally demonstrate a significant reduction in the healing time of iFSGs compared to collagen skin grafts (ProHeal^®^ Collagen Wound Dressing, MedSkin Solutions, Billerbeck, Germany) [31].

Other surgical wounds involve intrabuccal use to correct mucogingival deformities around teeth or promote granulation in a dehiscent abdominal wound before autologous mesh skin grafting [25,26].

Apart from wounds in adult patients, pediatric wounds have been treated with iFSGs, including neonatal calcinosis and acute wounds, due to different etiologies, such as animal bites, pressure ulcers or autoimmune disorders [27,28]. As in adults, experiences have been auspicious, reducing the expected healing time by about half [27].

Furthermore, iFSGs were originally developed to treat dermal and epidermal wounds and are not yet indicated for repairs where load-bearing support from the mesh is required, such as in the repair of any hernia. Intraperitoneal organ contact or bridging defects are further clinical contraindications up to date. However, due to the observed wound-healing effect in different wound types, more and more off-label uses are discussed in expert meetings. Non-published reports of surgeons convinced of the fast promotion of granulation and antibacterial resistance include using iFSGs to fill dead spaces in the body, such as the groin, in high-risk patients after vascular procedures or amputation stumps. Others have used iFSGs for wrapping around intestinal anastomosis or pancreatic-duodenal anastomoses for leakage prevention or for coverage of proximal abdominal aortic graft anastomosis to prevent aorto-duodenal fistula and graft infection. However, those off-label uses are currently not supported by evidence and only represent ideas of how the clinical indication of iFSGs could be extended in the future.

While iFSGs show significant clinical potential to improve general wound care, some challenges and considerations should be noted: the distribution and availability of iFSGs vary across different regions or healthcare facilities, potentially limiting their widespread use. However, distribution will undoubtedly change along with the development of the manufacturer’s infrastructure and its recently acclaimed acquisition by Coloplast, Humlebaek, Denmark. The high cost of iFSGs may be another limiting limiting factor known for other tissue-based therapies tissue-based therapies, especially in resource-limited settings or areas with inadequate healthcare coverage. However, a recently published cost analysis comparing iFSGs versus the SOC using collagen alginate therapy in diabetic foot ulcers demonstrated a reduction of the overall annualized treatment cost due to faster wound healing [47]. This analysis might convince healthcare providers to cover the treatment costs and support using iFSGs in every healthcare region independent of the local socioeconomic situation. Still, cost analyses for other indications that already require shorter healing times under the SOC are required to generalize this statement.

Moreover, more high-quality clinical studies with larger sample sizes are needed to provide more substantial evidence and guidelines for its use in NF and other wound etiologies. Although RCTs for diabetic foot ulcers are published, or in progress, there are no studies for other important wound etiologies, such as arterial or venous ulcers or burns. In addition, comparisons with other advanced wound techniques besides a SOC for these etiologies still need to be made.

## 5. Conclusions

The iFSGs represent a promising development in wound healing, with successful applications in the treatment of NF. Its biocompatibility, antimicrobial properties, and ability to promote tissue regeneration make it a compelling alternative or adjunctive therapy for this challenging condition, especially for treating extensive tissue loss after debridement. However, further research and the collection of clinical data are necessary to establish its effectiveness and fully address the potential limitations. With continued advancements in wound healing technology, iFSGs promise to improve patient outcomes and might revolutionize the management of wounds associated with NF.

## Figures and Tables

**Table 1:** Clinical spectrum of intact fish skin grafts published in the literature.

**Chronic Wounds** Diabetes mellitus/Diabetic foot syndrome [15,17,18,19,20,21,22,23,24,36]; Chronic Venous Insufficiency/Venous ulcers [15,17,23,24,37]; Peripheral arterial occlusive disease/Arterial ulcers [15,17].
**Atypical Wounds** Vasculitis (MPO (myeloperoxidase), p-ANCA, cryo-positive) of small, medium and large vessels in the setting of undifferentiated collagenosis [15]; Ischemic necrosis (sepsis-related, dialysis shunt-associated steal syndrome) [15]; Necrotic angiodermatitis [35]; Calciphylaxis [34]; Hemophilia-associated chronic wound [33].
**Burns** Partial thickness burns [24,29,32]; Full-thickness burns and blast areas [38]; Thin-skin graft donor site [29,30,31].
**Pediatric Patients** Neonatal calcinosis [28]; Acute wounds (animal bite, posttraumatic, sacral pressure ulcer, surgical dehiscence, autoimmune disorder) [27].
**Other Surgical Wounds** Dehisced abdominal wound [26]; Mucogingival deformities [25].
**Acute Traumatic wounds** [6,16,24,38]
**Necrotizing Fasciitis** (own experience)

## Data Availability

Data sharing not applicable.
